# The Glymphatic System: a Potential Key Player in Bacterial Meningitis

**DOI:** 10.1128/mbio.02350-22

**Published:** 2022-10-26

**Authors:** Marco Rinaldo Oggioni, Uwe Koedel

**Affiliations:** a Dipartimento di Farmacia e Biotecnologie, Università di Bologna, Bologna, Italy; b Department of Genetics and Genome Biology, University of Leicester, Leicester, United Kingdom; c Laboratory of Neuroinfectious Diseases, Department of Neurology, Klinikum Großhadern, Ludwig-Maximilians University, Munich, Germany

**Keywords:** *Streptococcus pneumoniae*, central nervous system infections, meningitis

## Abstract

The glial-lymphatic system (glymphatic system) is a recently characterized fluid clearance pathway of the central nervous system. Glymphatic system disfunctions leading to defects in drainage of the cerebrospinal fluid have been associated with several neurological disorders. In their article, J. S. Generoso, S. Thorsdottir, A. Collodel, R. R. E. Santo, et al. (mBio 13:e01886-22, 2022, https://doi.org/10.1128/mBio.01886-22) have now associated impaired glymphatic system functionality to neurological sequelae of murine meningitis caused by Streptococcus pneumoniae. Their work provides an initial and important step into the systematic evaluation of a potential impact of glymphatic system functionality on disease severity and sequelae in meningitis.

## COMMENTARY

The central nervous system (CNS) was long believed to be devoid of a lymphatic system. In the past decade, evidence for a highly organized fluid clearance pathway in the CNS called the glymphatic system (glial-lymphatic system) has grown (1). According to the glymphatic system concept—which has been reviewed extensively by researchers in favor for it (1) or in opposition to it (2)—cerebrospinal fluid (CSF) travels through periarterial spaces from the subarachnoid space to deeper brain regions, flowing into the brain parenchyma through aquaporin 4 (AQP4) channels located in astrocyte endfeet (3, 4). There, CSF is exchanged with interstitial fluid (ISF). The CSF-ISF mixture is then flushed out along perivenous spaces before exiting the CNS via lymphatic vessels located in the meninges and along cranial nerves, which drain into cervical lymph nodes and finally back into the venous system (5–7). The glymphatic system is considered to enable nutrient delivery to and waste elimination from the brain. Consequently, dysfunction of the glymphatic system can lead to the accumulation of toxic substances in the brain and thus can contribute to the pathogenesis of a variety of neurological disorders, such as Alzheimer disease, Parkinson’s disease, traumatic brain injury, ischemic stroke, and mood disorders (1, 8, 9). In the current issue of *mBio*, Generoso and colleagues (10) pursued the hypothesis that pneumococcal meningitis leads to impaired glymphatic system functionality, decreasing neurotoxic waste clearance in the brain and thus increasing inflammation and brain dysfunction. Using an established rat meningitis model with combined intracisternal injection of serotype 3 Streptococcus pneumoniae (control animals received artificial CSF instead) and the dye tracer Evans blue, the authors detected decreased Evans blue levels in serum samples but increased Evans blue levels in brains, especially in the external layer of the subarachnoid space and the cerebellum in meningitic rats compared to control animals, indicating an impaired drainage of the dye from the CNS into the circulation. As suggested by subsequent immunochemical analysis, this phenomenon was attributable to a progressive retraction of astrocyte endfeet from the vascular endothelium and consequently a misplacement of AQP4 channels. These pathological alterations were paralleled by a significant increase of bacterial products like the pneumococcal toxin pneumolysin (PLY) in the CSF (but not the brain parenchyma) over time, which in turn was associated with increased microglial activation, brain damage, and memory impairment. Overall, the study points to a new pathomechanism in pneumococcal meningitis, although some aspects require further investigation. For example, besides the glymphatic-meningeal lymphatic system, there seem to be other CSF outflow pathways, namely, the arachnoid villi, which are constructed as one-way valves permitting CSF and solutes to cross into the dural sinuses and thus straight into the blood (11). Therefore, additional examination of cervical lymph nodes for the presence of the dye tracer would be helpful to confirm the relevance of the glymphatic-lymphatic pathway. If there are clear differences in the Evans blue content between lymph nodes from meningitic and control rats, this would support the authors’ conclusion. In this context, it would also be particularly interesting to know what impact distinct manipulations of the glymphatic system activity have on the retention of Evans blue in the CSF as well as the course of pneumococcal meningitis. Did a reduction of glymphatic activity via, e.g., acetazolamide treatment, increase brain Evans blue concentrations during experimental pneumococcal meningitis (11)? In addition, what are the effects of drugs like dobutamine or norepinephrine receptor antagonists known to increase CSF influx into the brain on tracer accumulation, brain inflammation, and brain dysfunction (12)? The neuromodulator norepinephrine is a known driver of arousal and has been proposed to contribute to suppression of glymphatic system function during wakefulness (12). *In vivo* imaging analysis demonstrated a reduction of the CSF influx into the brain by ~90% in the awake state compared to anesthesia or sleep (13). It would therefore be exciting to see whether the course of pneumococcal meningitis can be positively influenced by permanent anesthesia. Besides wakefulness, the activity of the glymphatic system decreases sharply during aging (14, 15). It is worth investigating whether age-related glymphatic dysfunction contributes to the poorer prognosis of pneumococcal meningitis in the elderly (16). The suppression of the glymphatic system in old age seems to be caused—at least partly—by a loss of vascular polarization of astrocytic AQP4 channels in reactive astrocytes in old brains, which means that AQP4 is no longer confined to astrocytic endfeet. A quite similar phenomenon was observed in brains of meningitic rats in the study by Generoso and colleagues. Genetic depletion of AQP4 in mice has been shown to elicit a substantial reduction of the clearance of proteins like amyloid β and phosphorylated tau, thus contributing to their accumulation and deposition in the brain, proposed key steps in the pathogenesis of Alzheimer’s and Parkinson’s diseases, respectively (17, 18). Somewhat surprising given the observations of Generoso and colleagues, AQP4 deficiency has been reported to be protective in experimental pneumococcal meningitis, by dramatically reducing brain swelling and the rise in intracranial pressure, thus improving disease outcome (19). To what extent the observed AQP4-glymphatic system dysfunction and the beneficial effect of AQP4 deficiency in pneumococcal meningitis fit together must be clarified in future studies. All in all, Generoso's study points to an intriguing new possible pathomechanism in pneumococcal meningitis, opening a new field of research where further investigation may allow to demonstrate its definitive relevance to immune regulation, brain damage, and the clinical outcome of this disease.
